# The influential factors for achieving universal health coverage in Iran: a multimethod study

**DOI:** 10.1186/s12913-021-06673-0

**Published:** 2021-07-22

**Authors:** Naser Derakhshani, Mohammadreza Maleki, Hamid Pourasghari, Saber Azami-Aghdash

**Affiliations:** 1 Department of Health Services Management, School of Health Management and Information Sciences, Iran University of Medical Sciences, Tehran, Iran; 2 Tabriz Health Services Management Research Center, Health Management and Safety Promotion Research Institute, Tabriz University of Medical Sciences, Tabriz, Iran

**Keywords:** Influential factors, Universal health coverage, Health system, Control knobs, Systematic review, Iran

## Abstract

**Background:**

The initial purpose of healthcare systems around the world is to promote and maintain the health of the population. Universal Health Coverage (UHC) is a new approach by which a healthcare system can reach its goals. World Health Organization (WHO) emphasized maximum population coverage, health service coverage, and financial protection, as three dimensions of UHC. In progress for achieving UHC, recognizing the influential factors allows us to accelerate such progress. Therefore, this study aimed to identify the influential factors to achieve UHC in Iran.

**Methods:**

This is a multi-method study was conducted in four phases: First, a systematic review of the literature was conducted to identify the factors in PubMed, Web of Science, Embase, Scopus, ProQuest, Cochrane library, and Science Direct databases, and hand searching google scholar search engine. For recognizing the unmentioned factors, a qualitative study consisting of one session of Focus Group Discussion (FGD) and five semi-structured interviews with experts was designed. The extracted factors were merged and categorized by round table discussion. Finally, the pre-categorized factors were refined and re-categorized under the health system’s control knobs framework during three expert panel sessions.

**Results:**

Finally, 33 studies were included. Eight hundred two factors were extracted through systematic review and 96 factors through FGD and interviews (totally, 898). After refining them by the experts’ panel, 105 factors were categorized within the control knob framework (financing 19, payment system7, Organization 23, regulation and supervision 33, Behavior 11, and Others 12). The majority of the identified factors were related to the “regulation and supervision” dimension, whilst the “payment system” entailed the fewest. The political commitment during political turmoil, excessive attention to the treatment, referral system, paying out of pocket(OOP) and protection against high costs, economic growth, sanctions, conflict of interests, weakness of the information system, prioritization of services, health system fragmented, lack of managerial support and lack of standard benefits packages were identified as the leading factors on the way to UHC.

**Conclusion:**

Considering the distinctive role of the context in policymaking, the identification of the factors affecting UHC accompanying by the countries’ experiences about UHC, can boost our speed toward it. Moreover, adopting a long-term plan toward UHC based on these factors and the robust implementation of it pave the way for Iran to achieve better outcomes comparing to their efforts.

**Supplementary Information:**

The online version contains supplementary material available at 10.1186/s12913-021-06673-0.

## Background

Universal Health Coverage (UHC) has been introduced as a viable solution to promote, restore, and/or maintain the health of the population in countries [1, 2]. UHC has been defined as a way to provide quality health services to the destined population based on their health needs without suffering financial hardship [1, 3–5]. Furthermore, UHC is a way of promoting the population’s quality of life while ensuring financial risk protection, equity, and access to essential and quality health services [6–11].

In the past two decades, along with some High-Income Countries (HICs) that have achieved UHC, there was a substantial increase in the number of Low and Middle-Income countries (LMICs) showing great endeavors to reach the state of the sample countries [12, 13]. As, in the coming decades, most Asian and African countries will be able to implement basic and effective plans to achieve UHC by acquiring sufficient abilities to provide their essential resources in the health system for their country [14, 15].

Iran is one of the countries that seek to reach UHC in the next coming decade. In Iran, both the public and private sectors provide health services. However, the public sector has the upper hand in this issue [16]. The public health sector in Iran provides primary, secondary, and tertiary health services. On the other hand, the private sector focuses mainly on secondary and tertiary health care in urban areas [17]. To improve the health system and move towards UHC, Iran designed and implemented the Health Reform Program (HRP) in 2014. According to this reform policy, eight programs have been introduced in three steps aiming at UHC. The first step was to decrease OOP payments, extending insurance coverage, and improving the quality of health services in target populations Second step was about providing all services, drugs, and equipment needed by the inpatient wards, and the third one was about updating the tariffs on medical services [18–20]. Generally, the final destination of HRP was to improve the health indicators, to ensure equity in the delivery of health services, and to reduce health costs. The results of HRP in the short term indicated a decline in catastrophic health costs [18]. The health status of Iranian has been improved during the last decade’s thanks to the health networks where primary healthcare was provided for both rural and urban populations; however, many weaknesses and challenges are threatening the health system, such as high degrees of OOP payments [21], limited financial resources, increase in unofficial payments to the physicians [22], lack of community participation in solving health problems [23, 24], financial constraints, lack of clarity in tariffs setting mechanisms [25], and difficulties affecting the system due to international sanctions against Iran [26, 27].

Having the capabilities and potential to do so, Iran can take benefit of the experiences of LMICs such as Turkey [28] and Thailand [29] that have achieved UHC previously. This country has greatly reduced its distance to UHC compared to 22 LMICs as it seeks to mobilize resources, carry out reforms in policies, and fulfill political commitments to this mean [2, 30, 31]. Although all countries pursue the same goal, the method and timings are different based on the structure and available resources of the countries as well as the unique factors affecting countries [9]. For instance, countries such as Germany and Japan had achieved UHC in 127 and 36 years according to their specific structure and challenges [32].

Universal health coverage, as a comprehensive and new policy that overwhelm all parts of the health system and even other sectors, is influenced by many factors, such as political sustainability [33, 34], economic growth [35, 36], fragmentation in the health system [37–39], etc. Therefore, it is vital to set up the influential factors within a framework/model, to ensure the applicability of the policy at different levels of the health system. Also, it is essential to identify these factors according to the country’s unique circumstances. Because the factors act as a role model for health system managers to have due policy-makings. Moreover, the introduction of such factors results in the accurate, transparent, and accountable form of resource allocation which is a bottleneck in health systems. Furthermore, it seems that presenting the factors identified in the form of a framework increases the applicability and usability of the framework by policymakers and senior managers not only in the health system of Iran but also in other countries. Therefore, this study aimed to identify the factors that can facilitate or hinder the speed of achieving UHC in Iran.

## Methods

This study was conducted in four phases: (1) systematic review of relevant studies; (2) Qualitative Study: (Focus Group Discussion (FGD) & semi-structured interview); (3) merging factors based on Framework-Analysis; (4) refining and finalizing the factors in control knob framework through expert panels.

### Phase 1: systematic review

A systematic review was conducted in accordance with Preferred Reporting Items for Systematic Reviews and Meta-analysis (PRISMA) [40].

#### Search strategy and information sources

The required data were gathered by searching on 1 August 2020 and the search was updated on April 18, 2021, in the following databases: PubMed, EMBASE, Scopus, ProQuest, ISI Web of Science, Cochrane Library, Science Direct, and hand searching through Google Scholar search engine. Also, for finding national studies on UHC, the SID and MagIran were searched in Persian. Each database was searched according to its strategy. World Health Organization (WHO) and World Bank (WB) databases that host reports related to UHC were also searched. The key search terms selection was done by experts and using the mesh, which included ‘universal health coverage, ‘universal coverage’, ‘universal healthcare coverage’, ‘universal health care coverage’, ‘UHC’ and Iran combined with “OR” Boolean in the title or abstract (Additional file 1). Also, manual search and reference tracking was used to extract additional relevant studies based on citations of the eligible articles and documents.

#### Eligibility criteria

There were no time restrictions. The publication language was restricted to English and Persian. All study reports, including reviews, case studies, reports, and original studies that were related to affecting factors the achieving of UHC in Iran were included in the study. Also, the abstracts of the papers presented at the seminars and conferences, news, and studies irrelevant to the study objectives were excluded from the study. All retrieved studies were screened independently by two authors (ND and MRM). Disagreements were resolved via discussion until the mutual agreement was achieved. Whenever it was not possible, a third author (SA-A) helped with reaching the consensus.

#### Quality appraisal

All articles after extraction from the databases using the keywords mentioned were evaluated by two authors using the Critical Appraisal Skills Program (CASP) checklist. This checklist has 10 questions; the first two questions are for screening and answered with “yes” and “no”. If “yes” is the answer to the first two questions, the article will continue to be evaluated. The evaluator for each of the 8 following questions, one of the three options “yes” score 3, “no” score 1, and “cannot say” score 2, was selected. The maximum score of articles was 24 and the minimum score was 8. Papers which scores were less than 16 were excluded from the study. Any disagreements between authors were resolved through discussing with a third investigator.

#### Data extraction

Data Extraction in this study was done in several steps. After removing duplicate studies, titles of all articles were reviewed and articles that were incompatible with the aims of the study were excluded. Subsequently, abstracts and full texts of the articles were studied, respectively, and studies that did not meet the inclusion criteria and had poor correlation with study aims were identified and excluded. Data were extracted according to the researcher-made data extraction form and entered into the designed table. At first, as a pilot for the data extraction form, the data of 5 papers were extracted and the deficiencies of the original form were eliminated. The whole process of the systematic review was carried out by two researchers independently and disputes were referred to as a third researcher. A list of relevant affecting factors was arrayed as the output of this phase.

### Phase 2: FGD and semi-structured interview

A qualitative study was conducted to deeply explore and analyze the factors affecting the achievement of UHC in Iran. To do so, focus group discussions and semi-structured interviews were held in this phase.

#### Sampling

By using a purposeful sampling method [41], experts in the fields of Health Policy (two people), Health Services Administration (four people), and Health Economics (two people), a top manager from the Ministry Of Health (MOH), and two people who have practical experience in the field of UHC were selected. Experts who did not have the eligibility criteria were excluded.

#### Including and excluding criteria for experts selecting

- Having at least 5 year an executive experience or scientific background in the health system
- Having at least two scientific articles related to UHC
- Having at least a master’s degree in the health field.

#### Data collection

The only FGD session was held in the department of health services management, school of health management and medical informatics, Tabriz Universities of Medical Science. FGD session was held by the coordinator and the meeting’s leader to direct the discussion (the duration of the FGD was 110 min). All of the session was recorded using a digital audio recorder and then written literally on paper so that the listed affecting factors were identified and categorized.

Also, semi-structured interviews were conducted with five experts in the field of UHC until information saturation was reached. The semi-structured interviews were conducted in the participant’s office. The duration of each interview varied between 38 to 55 min. The participants’ statements were recorded by using a digital audio recorder and the research also used note-taking during the interviews. The interviews after the end of each interview were reviewed by the researchers several times and typed in Microsoft Word:2016. Data collection was continued until data saturation. In this study, after conducting four interviews, the researchers felt that the data was saturated. But for more certainty, another interview was conducted.

#### Data analysis

For data analysis, the content-analysis method was applied, which is a method to identify, analyze and report patterns (themes) within the text. This type of analysis is used when the theories on the subject are limited [42, 43]. Data analysis and coding processes were as follows: familiarizing with the data text, identifying and extracting the basic codes, identifying themes, reviewing and completing the identified themes, naming and defining themes, recoding and renaming some classes and themes, and ensuring the reliability of the codes.

#### Rigour

Responded validity was used to ensure the rigor and accuracy of the results. The participants’ statements were summarized at the end of each interview and FGD session, the participants were told to confirm the accuracy of the results.

### Phase 3: merging factors based on framework-analysis

In this phase, the factors identified in the previous stages were merged and duplicates factors removed through the research team round table. The initial draft of categorization was refined through research team meetings.

In this phase, the health system control knob [44] was used as a framework for categorizing factors UHC in Iran. Qualitative framework analysis is a flexible data analysis method that can be used in systematic qualitative reviews [45, 46]. The analysis process was started by studying the findings and making evidence-based inferences about organizing codes. The framework was designed and recommended in 2004 to improve the performance of countries’ health systems. Control knobs are used to determine the results and outputs of health systems to which it concentrates on five dimensions including, financing, payment system, organization, regulation & supervision, and behavior [44]. Considering that the control knobs framework was approved by the WHO and this framework considers the health systems from different points of view. One of the main reasons for deploying this framework was to identify areas of policy action to modify health systems and to improve their performance. Also, the main goals of health systems pursued by this framework (health status, customer satisfaction, and risk protection) are very similar to the dimensions of UHC. On the other hand, the intermediate goals of this framework (access, quality, and efficiency) somehow represent the goals of UHC. Another reason for using this framework is to influence the behavior of providers and consumers through population-based interventions in the health systems, which is a significant positive point in achieving UHC. Also, we created another column in this framework to categorize some factors that were not included in the health system control knobs columns.

Categorizing were done by two researchers (ND and MR.M) using the following steps:

1. Familiarization with data (reading selected factors extracted from previous phases);
2. Generating and restate clarify themes;
3. Classifying extracted factors based on content relationship and conceptual proximity;
4. Reviewing themes, merged and categories themes into the health system control knob columns;
5. Generating refined and clear definitions for themes in the health system control knob framework.

### Phase 4: expert panel

After the categorization affecting factors based on the health system control knob framework was refined through the research team, the draft version of the categorization was entered in the final phase.

The expert panel was conducted in three face-to-face sessions with the coordinator and meetings leader to control the discussion in the meantime of 65–90- min. One foreign country expert for research team consultation and poll on the categorization of factors in the framework through email and 5 local Iranian experts in UHC reviewed and discussed the content (as well as the face validity) of the health system control knob framework.

The opinions of experts were recorded by using a digital audio recorder and used by the research team to merge, add, and remove affecting factors in the health system control knob framework. Additionally, comments were obtained by the international expert were considered through the research team.

Also, for rigor in this phases, data transferability and reliability were used from peer review, expert check, and immersed.

To observe ethical issues in this study, all experts involved in the study were asked to fill out an informed consent form to participate in the FGD, semi-structured interview, and expert panel. This study was part of a Ph.D. Thesis supported by Iran University of Medical Sciences (Thesis NO: **19114**, ethical code: **IR.IUMS.REC.1399.674**).

## Results

**Fig. 1:**
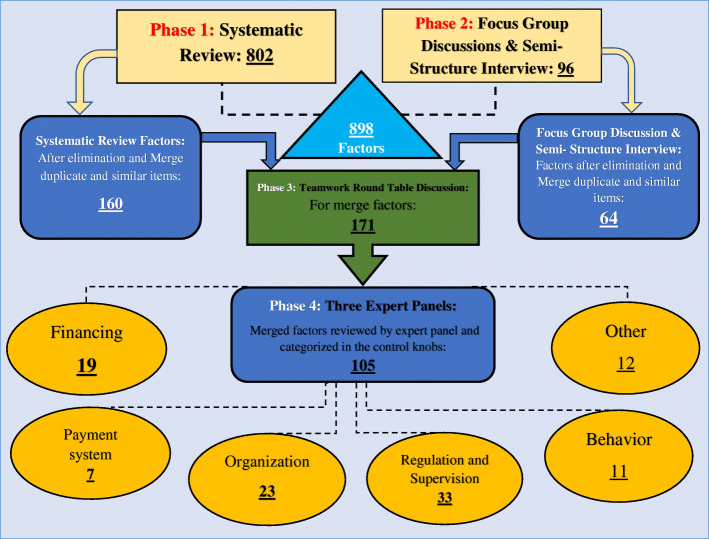
The four phases (systematic review, FGD, and semi-structured interview, merging Factors, and Expert Panel) of study and their results

The phases of the study (all four phases) and their results for a better understanding of the readers are presented in Fig. 1.

### Results of phase 1: systematic review

**Fig. 2:**
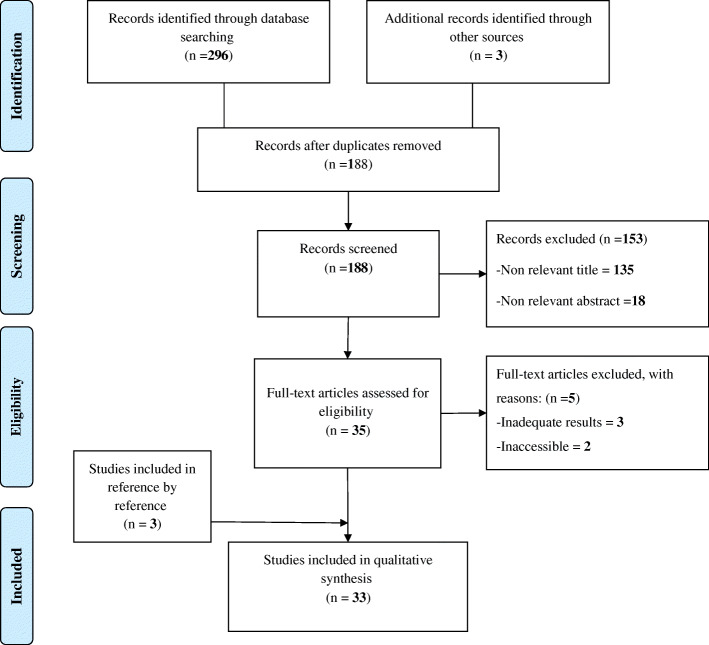
Flow diagram of the searches and Inclusion process

In this study, 296 articles were extracted from the databases and other sources, 108 articles were duplicates. Another 153 records were excluded by screening the title and abstract and 5 articles were removed due to lack of appropriate information and lack of reporting of the required information. Finally, 33 articles were included in the study [47–79] (Fig. 2 & Additional file 2).

In this phase, 802 factors were extracted. Finally, after the elimination and merge of duplicate and similar factors through the research team, factors were reduced to 160 factors (Additional file 3).

### Result of phase 2: FGD and semi-structured interviews

FGD and semi-structured interviews were done with the participation of the 16 eligible experts. In this phase, 96 extra factors were identified. Finally, after elimination and merge similar factors, factors were reduced to 64 factors (Additional file 4).

### Result of phases 3 and 4: merging factors and expert panel

In this phase, finalized affecting factors in phases 1 and 2 (160 factors through systematic review and 64 through FGD and semi-structured interviews), after elimination and merge duplicate and similar factors in round table discussion were reduced to 171 factors. Factors were classified into five dimensions related to the health system control knob framework and one another column. Finally, factors categorized in the framework axes were reviewed by expert panels and international experts and were reduced to 105 factors (Table 1).

**Table 1:** Categorization of extracted factors based on an expert panel on Health system Control knob in Iran

Financing	Payment system	Organization	Regulation and Supervision	Behavior	Others
▪ Financial risk protection ▪ Constraints and Structural barriers in Financial ▪ Methods of health system financing (specific tax to health, Sell resources, …) ▪ Sustainability of financing ▪ Health expenditure as % of GDP ▪ Out of pocket ▪ Method of Collecting ▪ Method of pooling money ▪ Strategic purchasing ▪ Health insurance system ▪ International assistance (donation, Charity) ▪ Benefit package ▪ Economic vulnerability in health ▪ International sanctions ▪ Costs Control ▪ Health service tariffs ▪ Per capita income ▪ The economic growth rate ▪ Inflation rate	▪ Exemption or Subsidies for prepayments ▪ Contribution-based on payment capacity ▪ Prepayment mechanisms ▪ Payment systems ▪ Information and interaction of insurance deductions for health ▪ Informal payments ▪ Deductible	▪ Health system Leadership ▪ Management in the health system (Resource management, human resources, Change management, …) ▪ Health infrastructure (technology, information system, …) ▪ The capacity of formulation and implementation of health policies ▪ Structural and functional reforms ▪ Distribution of health provider ▪ Decentralization in decision-making ▪ Non-governmental organizations (Civil society organizations: Private sector, NGOs and charities) participant ▪ Integration or Fragmented degree of the health system ▪ Equity in the distribution of health system resources ▪ Equity in access to health services ▪ Use of Appropriate technology in the health system ▪ The necessity for grading health service centers and giving the insured sufficient notice of this grading ▪ Bureaucratic obstacles ▪ Systematic perspective ▪ Inter and intra-sectoral collaboration ▪ teamwork ▪ Competency and Stability Management ▪ Policies and programs belonging to persons ▪ Effective Services Coverage ▪ Priority health services ▪ Overlaps in healthcare provision ▪ Involving all relevant stakeholders in the policy-making process	▪ Health system efficiency ▪ Government commitment ▪ Have Legal commitment ▪ Problems of law ▪ Political commitment and not having politically look ▪ Good governance ▪ Hasty policy implementation by politicians ▪ Conflict of interest ▪ Quality of health care services ▪ Supporting revision projects and national health indicators development. ▪ Focus on, villagers, nomads, less populated cities poor, disadvantaged and marginalized groups ▪ Family Physician Program ▪ Referral system ▪ Strengthen the central government’s Ministry of Health ▪ Control demands ▪ Regular transparency of revenues, expenditures, and activities ▪ Implement the rules of the World Health Organization ▪ Administrative and employment regulation ▪ Regulate the market of medical equipment ▪ Reviewing job classification schemes according to the needs of the health system. ▪ Electronic Health Record (EHR) ▪ Overlap in population coverage ▪ The dual practice of physician and another health workforce ▪ Competitive space between the providers. ▪ Policy dynamism ▪ Use of clinical guidelines and standards ▪ Performance of Supreme Council of Insurance ▪ oversight parliament ▪ Supervision by the ministry and the university ▪ The presence of specialists in public hospitals ▪ Plan to support the retention of physicians in underserved areas ▪ Assessment and accreditation of the health system performance ▪ Evidence-based policymaking	▪ Health promotion and education ▪ Culture-building ▪ Empowering community ▪ Perceived behavioral control ▪ Issues of urbanization ▪ Absence of obligation for health providers to contract with insurance organizations ▪ Negligence of social factors ▪ Social acceptability of health service ▪ public participation in health promotions programs ▪ Creating an incentive mechanism for behavior change ▪ The pattern of health service utilization	▪ Poverty ▪ Reviewing other countries experiences ▪ The unemployment rate in the country ▪ Active primary health care ▪ International relationship ▪ Health status of health indicators ▪ Prevention and control plans of non-communicable and communicable diseases ▪ Demographic and epidemiologic transitions ▪ Provide community-based services ▪ Health system service preferences (prevention-oriented or treatment-oriented) ▪ Disease Pattern ▪ Knowledge translation

**Fig. 3:**
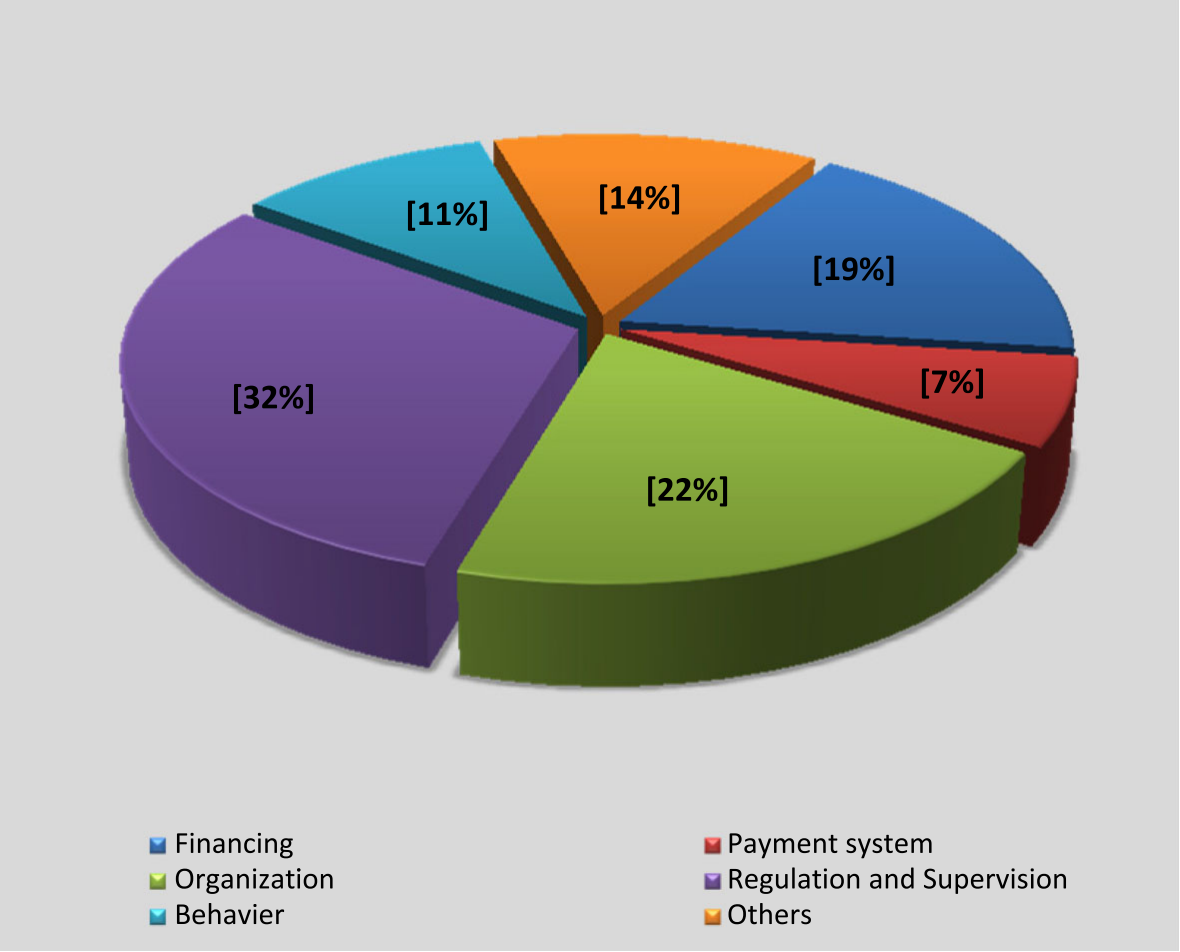
The percentage of influential factors for achieving universal health coverage in Iran based on health system control knob framework

In the last step, the percentage of each dimension of the health system control knob framework was calculated based on the frequency of factors. According to the results, regulation and supervision with (30%) have highest and payment system with (7%) has the lowest percentage (Fig. 3).

## Discussion

The present study was conducted in four consecutive stages to identify the factors in the progress towards UHC for Iran. ​In the systematic review stage, 160 factors and in the qualitative stage 64 factors were identified. In the third stage of the study, the factors were integrated and reduced to 171 factors. Finally, these factors through an expert panel were reduced to 105 factors and were classified within the health system’s control knobs framework.

We tried to provide a comprehensive and practical exploration of factors effective in achieving UHC. In recent years, after the introduction of UHC by the WHO, many international organizations and countries have tried to use such frameworks and models to achieve UHC. The WHO has also introduced some frameworks and models for this purpose [80]. One of these models presented by the WHO in 2017 was the framework of “strengthening health systems to achieve UHC”. In this framework, 4 main themes (governance and financial team, integrated service delivery team, health workforce development team, and essential medicines and technologies team), 16 dimensions, and 46 indicators have been introduced [81]. Certainly, combining and integrating the strengths of these frameworks and covering the weaknesses of each of them would be very helpful. Accordingly, after the identification and integration of the factors, the research team employed the framework of control knobs to classify and present the results. Policymakers and managers can use this framework as a useful and practical tool in achieving UHC, depending on the goals of their country’s health system [24].

Given the importance of “Financing”, not only the WHO considers it as one of the most important requirements for reaching UHC, but also necessitates countries to review their health financing policies [2, 82, 83]. The protection against financial risks in the UHC, an issue that is also mentioned in the framework of control knobs, is highly influenced by the financing of the country’s health system. In such a way, that choosing any of the financing methods by countries can have direct and indirect effects on the implementation of UHC [8, 10, 13, 31, 84, 85]. However, focusing on increasing economic growth [86, 87] and GDP, while allocating a proportional percentage of this GDP to the health sector are other leading elements in this case [83, 88]. Thus far, five influential factors were introduced which mostly covered the issues and aspects of financing in health systems. Due to the limitations in financing policies in different countries, these factors can act as leverage to improve financing policy-making and solve economic problems affecting this area. Some factors have played a more important role in financing the health system for achieving UHC, such as out-of-pocket payments, type of financing in the health system, high medical tariffs, sustainability of financing, international sanctions, service packages, strategic purchases, excessive inflation, and Health insurance system. A study in Lao PDR showed that to achieve UHC, the country needs health insurance and public awareness of health insurance and its benefits [89]. Also, Studies in LMICs show that high OOP payments can create a major gap in countries for Achieving UHC [90–94]. On the way to UHC, LMICs Governments require adequate and sustainable funding [91, 95, 96]. Commonly, governments use a combination of health financing mechanisms for financing, while LMICs rely more on OOP payments, social health insurance, international donor, and allocations from national budgets [95, 96]. A study conducted in seven LMICs (Costa Rica, Georgia, India, Malawi, Nigeria, Tanzania, and Thailand) showed that the health system financing through taxes and compulsory health insurance is very important to improve financial protection by reducing OOP payments [97]. also, capacity constraints in purchasing organizations are another problem that needs to be addressed in LMICs [97].

The “Payment System” is another health system’s control knob. Despite having fewer factors categorized within this dimension, the payment system plays a distinct role regarding UHC. The importance of this dimension best highlights when we consider its direct impact on three goals of UHC including, financial protection, population coverage, and service coverage. Also, it can affect justice in access to health services and make the path clear or uneven to UHC for every country [98–100]. It should be noted that the payment system is affected by other control knobs such as financing, organizing and legislation, and supervision, and is adjusted according to these knobs. This can make this control knob more powerful and turn it into an executive arm (stakeholder control system) among the control knobs. Successful implementation and monitoring of the implementation of this control knob will be crucial for any country to reach UHC [101, 102]. The Philippines has shifted the payment mechanism away from fee for services to case-based payments, which can be chivied to the goals such as; streamlining claims payment, increasing transparency, optimizing health care delivery, and ultimately achieving greater financial support [103, 104].

Proper service coverage for the people of any country requires due organization of the health system to pave the way for achieving UHC. The control knob of “Organization”, with 23 factors, plays a decisive and powerful role in achieving UHC. Paying attention to the characteristics of this knob, enables policy-makers and managers to establish internal and external strong relationships, manage the resources effectively, and provide a proper distribution of resources based on the capacity and infrastructure of each society that can bring the country one step closer to UHC [51, 59, 105–108]. Furthermore, through a systematic perspective on the health system, this dimension can lead to the unity of the health system, and by eliminating organizational and managerial barriers, it provides fair access to health services for people [109–111]. In LMICs, this dimension takes a double-folded value, where, the countries face severe shortages in health resources [58, 112–116].

The study indicated that the most number of identified factors were within “the Regulation and Supervision” control knob (33 factors). As mentioned, UHC is a new philosophy and approach, making major changes in most parts of the health system and other sectors related to the health system. Therefore, a coherent and effective regulation and supervision system must be established at the beginning of the path to achieving this goal. Adequate supervision can ensure a legal context to strengthen the health system which results in due employment of policies and regulations to reach UHC [117–119]. As mentioned before, there are fundamental problems and deficiencies in LMICs in terms of weak legislation and regulatory structures that demand to strengthen this knob through effectively dealing with the factors introduced during the current study. Another critical point is the great dependence of this knob on the context of the health system which contains the political and administrative structure of the country, too. This is because of various laws and miscellaneous methods of monitoring that are developed and applied by external regulators. Therefore, it necessitates paying a sufficient amount for inter-sectoral partnerships and government commitment in this dimension. Also, the case studies in LMICs highlighted the critical roles of political leadership, political commitment, and good governance in pursuing and sustaining UHC policies [97, 120–123]. Lack of political commitment and commitment to UHC was identified as a common challenge among African countries [124]. A study conducted in 11 different countries (Bangladesh, Brazil, Ethiopia, France, Ghana, Indonesia, Japan, Peru, Thailand, Turkey, and Vietnam) with different UHC status showed that countries must have both of the necessary technical and political knowledge to move towards UHC [125].

The last control knob in the framework “behavior”. This knob is associated with all stakeholders in the health system and is effective in regulating the behavior of the provider, recipient, and buyer of the service. Culture building and attracting public participation are two crucial elements of this dimension [126–128] in achieving the goals of the health system. People’s participation can provide a constant movement towards UHC [119, 129]. This knob, by coordinating stakeholders and involving them, can act as a facilitator and accelerator bringing the country closer to the UHC in less time. As mentioned, community participation has a distinct role regarding societies’ health behaviors, therefore, the concentration of the health system should be the promotion of social culture and public health. The most beneficial measures could be the proper use of health resources, promoting health behaviors, improving health literacy, promoting the culture of self-care, and empowering people to participate in strengthening and supporting the health system.

In a study entitled “the challenges and opportunities to achieving UHC in Nepal”, the factors such as using Constitutional provision, global support, and progress on the health insurance act, decentralization of health service to the grass-roots level, were introduced as an opportunity to move towards UHC. Also, the support of stakeholders, a sense of national priority and international support, Government stewardship, the political commitment under the changing political context, and fair contribution and distribution of resources by appropriate health financing modality as facilitators of the country’s health system would boost the speed of achieving UHC in Nepal [130]. In another study in China, factors such as social security system, population, economic development, and education have been introduced as contextual factors for achieving UHC [131]. Moreover, factors such as lack of capacity service, socioeconomic disparities in the access to and utilization of hospital-level health services and costs, out-of-pocket spending, the low-income context in the country, harness the political commitment, and geographical barriers have been introduced factors for achieving UHC in Ethiopia [132].

In the present study, some factors that could not be classified in the control knobs were categorized in a separate column and were discussed between experts. Items such as poverty, review of other countries’ experiences, unemployment rate, active delivery of primary health care, the status of the country’s health indicators, demographic and epidemiological transition, knowledge transfer, community-based service delivery, health system service preferences (prevention-oriented or treatment-oriented), diseases pattern and communicable and non-communicable disease prevention programs, were categorized in this column. Given the importance of these factors, the researchers did not rule out these cases. Therefore, it seems that these cases can play a complementary role to other control knobs.

## Limitation

One of the limitations of the present study was the participation of only Iranian experts and also the systematic review which was limited to research conducted in Iran, which could reduce the generalizability of the results. To increase the generalizability and validity of the study results, the researchers tried to identify and categorize the factors using several different methods (systematic review, a qualitative study including focused group discussion and semi-structured interview, expert panel, and teamwork). Study design in this way can increase the usability of the study results and their validity. Also, to increase the generalizability of the results, in this study, the framework of the control knobs of the WHO has been used to classify the factors. This feature of the study can also allow other countries to benefit from the results of the study.

## Conclusion

The results of the study show that in achieving UHC, countries need to identify the influencing factors and prioritize these factors following their internal capacities. Categorizing the influencing factors in achieving UHC in the form of an approved framework such as control knobs can give countries the advantage of having a comprehensive analysis of their health system. Therefore, considering these factors, countries can identify their strengths and weaknesses in this path and design the necessary and planned measures in this direction to accelerate the achievement of UHC.

This study, by presenting the factors and classifying them in the framework of control knobs, facilitates the recognition of these factors according to their nature and the main area for countries and provides the basis for measures to correct and cover weaknesses in the health system of countries.

## Supplementary Information

**Additional file 1.** Appendix 1- Complete search strategies for databases.

**Additional file 2.** Appendix 2- Characteristics of extracted studies with consideration to affecting factors to achieving UHC in Iran.

**Additional file 3.** Appendix 3- Identification affecting factors to achieving UHC trough systematic review in Iran.

**Additional file 4.** Appendix 4- Identification affecting factors to achieving UHC trough FGD and semi-structured interviews in Iran.

**Additional file 5.** Appendix 5- List of excluded studies.

**Additional file 6.** Appendix 6- Quality assessment of the study.

## Data Availability

All data generated or analyzed during this study are included in this published article in Additional files 1 and 2.

## Abbreviations

UHC: Universal Health Coverage
OOP: Out Of Pocket
LMICs: Low and Middle-Income Countries
HICs: High-Income Countries
WHO: World Health Organization
WB: World Bank
FGD: Focus Group Discussion
CASP: Critical Appraisal Skills Program
LIC: Low-Income Countries
