# Supplementary material for: The influential factors for achieving universal health coverage in Iran: a multimethod study
Source: BMC Health Serv Res. 2021 Jul 22;21:724. doi: 10.1186/s12913-021-06673-0 (PMC8299681; doi:10.1186/s12913-021-06673-0)
Supplement: Supplementary file 1 — Additional file 1. Appendix 1- Complete search strategies for databases. [file 12913_2021_6673_MOESM1_ESM.docx]

| **Appendix 1**: Complete search strategy for **PubMed** databases | | |
| --- | --- | --- |
| **Set** | **Strategy** | **Results** |
| **#1** | (((("universal health coverage"[Title/Abstract]) OR ("universal healthcare coverage"[Title/Abstract])) OR ("universal health care coverage"[Title/Abstract])) OR ("universal coverage"[Title/Abstract])) OR (UHC[Title/Abstract]) | 5132 |
| **#2** | Iran [Title/Abstract] | 42599 |
| **#3** | #1 AND #2 | 46 |

| **Appendix 1**: Complete search strategy for **Scopus** databases | | |
| --- | --- | --- |
| **Set** | **Strategy** | **Results** |
| **#1** | ( TITLE-ABS-KEY ( "universal health coverage" ) OR TITLE-ABS-KEY ( "universal healthcare coverage" ) OR TITLE-ABS-KEY ( "universal health care coverage" ) OR TITLE-ABS-KEY ( "universal coverage" ) OR TITLE-ABS-KEY ( UHC ) ) | 8854 |
| **#2** | TITLE-ABS-KEY ( Iran ) | 143081 |
| **#3** | #1 AND #2 | 74 |

| **Appendix 1**: Complete search strategy for **Web of Sciences** databases | | |
| --- | --- | --- |
| **Set** | **Strategy** | **Results** |
| **#1** | TOPIC: ("universal health coverage") OR TOPIC: ("universal health care coverage") OR TOPIC: ("universal healthcare coverage") OR TOPIC: ("universal coverage") OR TOPIC: (UHC)  Refined by: LANGUAGES: ( ENGLISH) | 5646 |
| **#2** | TOPIC: (Iran) | 94033 |
| **#3** | #1 AND #2 | 56 |

| **Appendix 1**: Complete search strategy for **Embase** databases | | |
| --- | --- | --- |
| **Set** | **Strategy** | **Results** |
| **#1** | 'universal health coverage':ti,ab,kw OR 'universal healthcare coverage':ti,ab,kw OR 'universal health care coverage':ti,ab,kw OR 'universal coverage':ti,ab,kw OR uhc:ti,ab,kw | 6120 |
| **#2** | Iran:ti,ab,kw | 59806 |
| **#3** | #1 AND #2 | 54 |

| **Appendix 1**: Complete search strategy for **ProQuest** databases | | |
| --- | --- | --- |
| **Set** | **Strategy** | **Results** |
| **#1** | ti("universal health coverage") OR ti("universal healthcare coverage") OR ti("universal health care coverage") OR ti("universal coverage") OR ti(UHC) | 349 |
| **#2** | ti(Iran) | 6248 |
| **#3** | #1 AND #2 | 4 |

| **Appendix 1**: Complete search strategy for **Cochrane** databases | | |
| --- | --- | --- |
| **Set** | **Strategy** | **Results** |
| **#1** | ("universal health coverage"):ti,ab,kw OR ("universal healthcare coverage"):ti,ab,kw OR ("universal health care coverage"):ti,ab,kw OR ("universal coverage"):ti,ab,kw OR (UHC):ti,ab,kw | 131 |
| **#2** | (Iran):ti,ab,kw | 7109 |
| **#3** | #1 AND #2 | 45 |

| **Appendix 1**: Complete search strategy for **Science Direct** databases | | |
| --- | --- | --- |
| **Set** | **Strategy** | **Results** |
| **#1** | ti,ab,kw: "universal health coverage" OR "universal healthcare coverage" OR "universal health care coverage" OR "universal coverage" OR UHC | 1349 |
| **#2** | ti,ab,kw: Iran | 21102 |
| **#3** | #1 AND #2 | 8 |
