# Supplementary material for: The influential factors for achieving universal health coverage in Iran: a multimethod study
Source: BMC Health Serv Res. 2021 Jul 22;21:724. doi: 10.1186/s12913-021-06673-0 (PMC8299681; doi:10.1186/s12913-021-06673-0)
Supplement: Supplementary file 2 — Additional file 2. Appendix 2- Characteristics of extracted studies with consideration to affecting factors to achieving UHC in Iran. [file 12913_2021_6673_MOESM2_ESM.docx]

| **Appendix 2-** Characteristics of extracted studies with consideration to affecting factors to achieving UHC in Iran | | | | |
| --- | --- | --- | --- | --- |
| Authors and year | Title | Document type | Method | Determinant factors |
| Peykari, N. et al, 2017 [33] | National action plan for non-communicable  diseases prevention and control in Iran; a response to emerging epidemic | National Action Plan | --- | - Financial risk protection - Effective attention to non-communicable diseases - The time bounded national action plan - Inter and intra-sectoral collaboration - Holistic political support and responsibility - Participation of sub-national policy makers - Leadership - Change management - Financial constraints - Health infrastructure - Inadequate qualified human resources - Poor information and communication - Coordination resistance of some organizations |
| Universal Health Coverage in Iran  2014 [34] | Universal Health Coverage in Iran Observatory report of Islamic republic of Iran's National Institute of Health Research | Report | systematic review &  Qualitative Study | ***Chapter 2:***   - Develop Effective Services Coverage in Iran - Training professional Human resources for assessing effective health services coverage. - Creating appropriate atmosphere to criticize findings of effective health services coverage assessments. - Developing a single website for information dissemination and access to required data for effective health services coverage assessments. - Devoting financial resources to support information collecting system launch. - Using national media for culture-building - Creating an incentive mechanism for successful universities and organizations to manage information - Creating integrated infrastructure for data collection at health ministry level - Supporting revision projects and national health indicators development. - Developing the reporting system on the basis of knowledge translation system. - Developing legal requirements concerning data collection and dissemination - Reviewing other countries experiences   ***Chapter 5:***   - Subsidies for prepayments - Tax-based financing - Stability of financing sources - High out of pocket payments - Payment exemption - Contribution based on payment capacity   ***Chapter 6:***   - Commitment to access all population groups - Equity in Access - Financial protection against catastrophic costs - The political and financial commitment - Civil society organizations - Technical capacity to produce - Use of research evidence - Fiscal policy space - Capacity building for learning from experience and accelerating the implementation - The long-term political commitment - Management - Out of pocket payment - Pre-Payment - Costs Control - Financing based on prepayment - Insurance - Focus on poor and marginalized groups - Development of the Family Health program - Accumulation and payment - Taxes - Participation mandatory health insurance - Income tax - International assistance - Development of payment systems - Integration of labor resources in health sector - The network communication facilities and health sector - The health care technology - The information systems in health sector - Quality - Strengthening electronic data recording - Strengthen the central government's Ministry of Health - Reduce the amount paid by patients in public hospitals - Plan to support the retention of physicians in underserved areas - Financial protection of specific diseases, incurable - Priority services - Distribution of resources - Poverty - Household financial contributions - Income - The risk of multi aggregation - The high administrative costs - Multiplicity of funds - The economic situation (high or low economic growth) - Government funds to the health sector Research - The high unemployment rate in the country - Inefficient and non-transparent tax system - The economic crisis - Weaknesses in inter sectoral collaboration - Failure to balance the interests of stakeholders in the health system - Attention to various aspects of economic, cultural and social programs and health interventions - Attention to the structures and substrates in the formulation and implementation of policies - Removal and installation of high management levels. - Policies and programs belonging to persons - Collect and accumulate - The position of the private sector in the provision of services - Training of human resources - Shortage of supply and skyrocketing medical staff - The lack of evidence-based management - TECHNOLOGY assessment of health - Stability Management - Appropriations stable budget from government - Development of clinical guidelines - Integration Fund - Family Physician Program - The long-term plan - Assessment of the status quo - Strengthening primary health care - The definition of a service package - Have Political commitment and not having politically look - Financial stability - Purchasing strategic - Legal commitment - Commitment to do - Tariff weak - Accurate information system - Removing Political gaps - Weak oversight Parliament - Functionally independent states - Treatment |
| Ibrahimipour, H, et al, 2011 [36] | A qualitative study of the difficulties in reaching sustainable universal health insurance coverage in Iran | Article | qualitative descriptive  cross-sectional | - Lack of information - Regressive financing and non-transparent financial flow - High rate of OOP payment - Fragmented and non-compulsory system (Fragmented pools) - Standard benefit package - Payment system - Supply management system - Managerial concerns - Governmental participation - Uncontrolled demands - Administrative deficiency - Lack of clear information on coverage |
| Piroozi, B, et al, 2015 [37] | Catastrophic Health Expenditure After the Implementation of Health Sector Evolution Plan: A Case Study in the West of Iran | Article | Qualitative Study | - Reduce the OOP health expenditures - Catastrophic health expenditures |
| Mehrolhasani, MH, et al, 2017 [38] | A Review of the Health Financing Policies Towards Universal Health Coverage in Iran | Article | Qualitative study | - Public health insurance - Adopt and implement effective equitable financing policies - Collect resources - Allocate resources - Resource management and accumulation - Strategic shopping - Commitment to established rules and policies - Insurance Funds Integration - Increase sustainable resources - Subsidized allocation of subsidies - Prioritize health services - Establishing and improving a performance-based payment system - Adequate political support - Compatibility of program design with infrastructure - Increasing the share of health in GDP and the government's general budget - Compulsory public health insurance - Establishing a suitable system for collecting insurance premiums - Imposition of taxes and duties in order to increase the income of the health sector - Targeting subsidies - Regular transparency of revenues, expenditures and activities - Increasing fundraising funds and controlling its effectiveness ** - Emphasis on financial stability over a period of time - Determining the comprehensive health care package - Provide standards of health care services based on health service leveling - Expanding air and ground emergency services coverage - Natural childbirth expansion program - Improving the quality and effectiveness of services - Establishment of health centers in the free zones - Improving the quality of quality services in public hospitals - Improving the quality of hoteling in public hospitals - Unconditional care and treatment of the injured in traffic accidents - The presence of specialists in public hospitals - The presence of doctors in less developed and deprived areas - Complete coverage of medical needs by basic and supplementary insurance - Strengthen a competitive market for insurance services - Improving the payment system to providers - Development of health service tariffs - High cost of out-of-pocket payments - Catastrophic expenditure - Strengthen the referral system - Ensuring the quality of services and meeting the needs of society - Improve management and planning in the health sector - Ensuring good governance in the health sector - Assigning part of the health care and cost control to the private sector - Conflict of interest - Lack of common understanding between stakeholders at different levels |
| Mousavi S M & Sadeghifar J, 2016 [39] | Universal health coverage in Iran | Correspondence |  | - OOP - Economic growth - Economic sanctions - Inequality in the distribution of health-care services - Concentration of hospitals in the large metropolises - Fragmentation of insurance funds - Incomplete population coverage - Overlap in population coverage - Inappropriate service coverage - Public dissatisfaction. - Sustainability of reforms (financial and managerial) - Managerial appointments in health sector should be based on meritocratic principles. - Adopt the intersectoral collaboration - Comprehensive measurements and monitoring of the progress |
| Moghadam MN, et al, 2012 [40] | Iranian Household Financial Protection against Catastrophic Health Care Expenditures |  | descriptive- analytical study cross sectional study | - Reducing catastrophic healthcare expenditure - Health insurance coverage - Different fee schedules practiced by private and public providers, - Referral system |
| Marnani AB, et al, 2012 [41] | Challenges of a Large Health Insurance Organization in Iran: A Qualitative Study | Article | Qualitative Study | - Increasing costs - The lack of comprehensiveness, adequacy and quality of services - The lack of clear boundaries between basic and supplementary health insurance packages - The problems on determining the scientific and fact-based tariffs - Financial constraints - The lack of clarity in the level of commitments and covered services in basic package - Lack of a prevention center in the current organizational structure - The mismatch between the organizational structure and updated information technology systems - Lack of an appropriate control system in entry of new services to the organization - Many structural changes - Structural barriers in establishing financial communication with and giving notice to centers through network - Organization's inability to achieve its missions - Shortage of skilled manpower at the headquarters - The mismatch between skilled human resources and organizational missions - 15.developing a system for participating people, centers under contract with the insurance organization, and stakeholders - Lack of a comprehensive and updated mechanized systems for recording insured-related statistics - Failure of information systems related to the insured - Shortage of skilled manpower in the field of prevention - Shortage of skilled manpower in the field of treatment - The mismatch between the structure and organizational missions - Entry of the new drugs to the supplementary services package without any expert working - Lack of access to desirable situation using available resources - Lack of appropriate training and instructions for the newcomers to the organization on arrival - Weaknesses in resource management - Weaknesses in the patient information system - Performance evaluation of managers regardless of their expert decisions - Making appropriate policies on outsourcing as a solution to the problems of overcrowding in the pay day branches - The problems of the implementation methods of outsourcing - The contradictions in the simultaneity of developing the insurance id and the health smart card - Decreased commitments to services due to growing costs - Lack of any indicator for grading health service centers and putting all of them at the same level - The necessity for grading health service centers and giving the insured sufficient notice of this grading - Working in parallel and the lack of integration in the insurance data - The need for integration and standardization of the ministry of health policies on health insurance - Completing supplementary insurance package and strengthening supportive insurance - Appropriate expansion of health services in border areas - The weak performance of supreme council of insurance - Absence of obligation for physicians to contract with insurance organizations - Lack of coordination between ministry of health and insurance organizations - Lack of any measure which is necessary for leading physicians' performance - Improper use patterns in the health sector - Insufficient service coverage in basic health insurance package - Lack of health services to purchase in some disadvantaged areas - The medical community's influences on policy and decision making in the health system - Lack of health ministry's domination on factors affecting health - The necessity of policy-making integration in the health system - Lack of coordination among decisions about health made by parliament, ministry of welfare and social security, and the ministry of health - People's freedom to refer to the physicians - Problems relating to setting tariffs and using unlicensed services in the country - Reluctance of some private centers for contracting - Taking a long time to receive required services in the pay-day branches - Overcrowding in the pay-day branches - Low level of the insured's information about the insurance organization services and the necessity of giving them appropriate notice - Lack of the insured's information about the similarity of services in the centers under contract with the private centers - Lack of the insured's information about the difficulty of the process of receiving costs - Lack of the insured's information about the insurance organization's commitments in the private sector - Lack of effectiveness of giving information to the insured using booklets - The necessity of creating a culture of the insurance organization control over physicians - Lack of patients' awareness of how to use insurance id accurately - Patients' indifference to detach the insurance id's sheets - The necessity of creating a culture of appropriate financial relationships among the insured, physicians and the insurance organization - The necessity of creating a culture of supervision in the organization between managers and employees - Being wrong to receive services for free. - The necessity of information and interaction of insurance deductions for heal |
| Abdellah Y, et al, 2019 [42] | The role of access to affordable and quality assured blood and blood products for achieving Universal Health Coverage | Letter to the Editor |  | - Proper investment in access to cheap and quality blood and blood products - Blood transfusion systems - Establish an effective and regular national service in blood supply and transfer - Sufficient funding |
| Bakhtiari A, et al, 2017 [43] | Design and deployment of health complexes in line with universal health coverage by focusing on the marginalized population in Tabriz, Iran | article | Qualitative method | - Implementing the health complex strategy in marginalized areas - Increasing the number of health care worker - Increasing the number of health institutions - Creating efficient, decentralized and integrated health systems - Trained and motivated staff - Provide quality health care services - Guaranteed financial resources - Reduce out-of-pocket payments - Improving health indicators - Increase customer satisfaction and service providers - Justice in health - Improve quality - Price control of health services - Improving the behavior of recipients and service providers - Correction of payment system and method of purchasing services - Identify the best structure and strategy for implementing health programs - Empowering managers and employees of the country's health sector - Completion, equipment and development of the country's health care network - Public and non-governmental sector participation and cooperation - Health service packages - Improving the information technology system, recording information and health statistics - Establishment of health monitoring and evaluation system - Collaborate with training centers to take advantage of their capacity - Establishment of a health monitoring system - Provide pre-hospital emergencies to villagers, nomads and less populated cities - Collaborate with research centers - In-service training for public and private forces - Use the potential of the private sector to provide matte health services - Pay close attention to prevention and diagnosis until treatment - Pay attention to the referral system - Family doctor's attention - Cost and structure management - Electronic Health Case - Physician training - Development of service leveling program and performance-based payment system - Pay attention to health instead of patient-centeredness |
| Alinia C and Davoodi Lahijan J, 2019 [44] | Moving Toward Universal Health Coverage: Four Decades of Experience from The Iranian Health System | Perspective |  | - ***Service delivery:*** - Low accountability - Inequity in utilization - Poor quality of care - Inducted demand - ***Sustainable resources:*** - Uncertain and dependent budget - Unpredictable of government commitment - Inefficient budget as percent of GDP - Strategic purchasing - Non-scientific basic package - Lack of cost effectiveness criteria’s - Payment systems - Governance - Explicit orientation to UHC - Intersectional cooperation - Evidence-based policy making - Stakeholder involvement - Resource production - Human resources management - Overutilization of drugs and equipment - Inefficient using of resources - Low income per capita - Low economic growth - High rate of unemployment - Inefficient and non-transparent tax system - Reducing the out-of-pocket expenditures - Health-care services package - Financial support - Scientific, transparent, and cost-effective priority-setting - Competitive space between the providers. - Monopoly power has made ineffective many of cost control policies - Integrating the multiple health insurances - Using scientific tools in resource allocation - How to spend the resources? - Referral system |
| E Ehsani-Chimeh, et al, 2018 [45] | Iran towards universal health coverage: The role of human resources for health |  |  | - Unified governance and comprehensive planning - Conflicts of interest - Rationalizing tariffs - Supporting the inhabitancy of physicians in deprived areas - Supporting the presence of specialists in the MOHME hospitals - Improving the quality of health care services - Recruiting full-time faculty staff - Performance-based payment - Implementing primary care and public health dimensions - Recruiting HRH - Revising the administrative and employment regulation - Developing a transformation plan for medical sciences - Training to enhance skills and empowering primary care managers and health service providers - Establishing a specialized company for outsourcing hrh - Reduction of compulsory services in deprived and less developed areas - Improve the quality of health workforce - Motivation among the workforce - Accreditation system in HRH - Comprehensive system for HRH management - Unequal geographical distribution of HRH - Inadequate and sometimes, contradictory laws, lack of unity in the implementation of laws, and existence of various interpretations from laws |
| Zalani, GS, et al, 2018 [46] | Human resources for health strategies: the way to achieve universal health coverage in the Islamic Republic of Iran |  | Qualitative study | - Advocacy among different levels - Systematic evaluation framework   ***Education pathway***   - Developing the required new disciplines, and balancing the entries to the majors with shortage or moderate the entries of excess majors. - Providing all necessary resources for the affiliated universities and units - Ensure the continuity of training - Supply staffing needs with the priority of recruiting qualified local hr - Review and update educational curriculums of courses - Promoting the position pyramid of full-time faculty members - Absorbing GP and other health graduates - Estimating HR requirements in different geographic regions - Accreditation of education system - Calculating the actual cost of diagnostic and medical services, tariffs and fees   ***Retention & incentives pathway***   - Pay extra amounts for severe weather conditions, hardship of job, shift working, being full-time and etc. - Calculating and financing the cost per capita for training the demanded hr - Recruiting the faculty members as full-time for at least 5 years   ***Skills mix pathway***   - Providing the necessary mechanisms to enhance the role and status of the central headquarter of MOHME. - To plan and implement continuous training at the staff & managerial level - Formulating necessary protocols to provide qualitative and quantitative hr - Reviewing job classification schemes according to the needs of the health system. - Task shifting and continually revising the professional boundaries and the skill mix   ***Labor markets pathway***   - Providing mechanisms for reviewing elites and specialist’s outflows from public to private sector - Introduce surplus workforces, to the applicant countries for their recruitment, in compliance with the who code of practice - To absorb health workforces from impoverished and low income countries   ***Cross-cutting strategies:***   - Establishing a “national committee for coordination, policy making and hrh planning” - Establishing and deploying “health sector hr observatory” - Allocating a suitable proportion of resources earned to hrh. - Designing and deploying the comprehensive information system for hrh - Delegating executive and outsourcing affairs to the private sector. |
| Emami Razavi SH. 2016 [47] | Health system reform plan in Iran: Approaching Universal Health Coverage | Letter to editor |  | - Reduce treatment costs - Expanding health insurance coverage. - Development of justice in the field of health - Review subsidy policies - Provide fair financial access - Insuring people without insurance - Coverage of medical services in public hospitals - Supporting people and providers of natural childbirth services - Support for incurable patients - Supporting residents and keeping doctors in hospitals and deprived areas - Improving the quality of hoteling in public hospitals - Primary health care for villagers - Development of primary health care services in cities - Completion, development and improvement of the family physician program - Referral system in urban areas - Develop and promote self-care and empowerment of the people - Strengthen inter-sectoral cooperation - Public Health Program - Transformation of oral health - Reduce public spending in the public and private sectors - Doctors' desktop payments - Pay out of pocket - Lower back costs - Correction of medical tariffs - Establish an additional control system for non-payment of fees - Performance-based payment system. - Increase efficiency and improve quality - Satisfying the treatment staff and clients - Futurism package and scientific authority in medical education - Package moving to third generation universities - Training package - Strategic Development Package - Education Internationalization Package - Package for the promotion of the evaluation system and medical sciences exams - Credit package for educational institutions and hospitals - Development package for improving the infrastructure of medical education - Lack of cooperation between insurance companies - Failure to reach maturity in hospital information system - Program support, both financially and politically, at high levels of decision-making - Integration of insurance organizations - Continuous monitoring of programs - Efficient manpower training |
| Riazi-Isfahani, S. et al, 2018 [48] | Universal health coverage in Iran: Health-related  intersectoral actions |  | Debate Article | - Health-related intersectoral actions - Intersectoral governance interventions - Continuity and sustainability of the policies and programs - Establishing provincial health policy secretariats (PHPSS) - Establishing a community-based council called “civil society participation house in health (CSPHH)” - Empowering people - Promoting health literacy - Producing practical instructions - Developing high-level policy documents based on SDH approach in provincial level called “provincial health plan” - Establishing health assemblies in different levels form local to national level, - Establishing a community-based center called “neighborhood health center” - Predesigned systematic monitoring system for council of health and food security (SCHFS) - Obligation of government - Passing the act of “permanent activities of SCHFS” in the parliament - Sectoral and intersectoral policymaking - Developing an intersectoral multilevel decision-making structure - Empowering the SCHFS - Health impact assessment (HIA) - Health charity assemblies in national and provincial levels - Allocating public fund resources of family physician programs to different geographical regions - Addressing gender equity in different terms - Passing the act of family protection by the parliament. - Addressing gender specific health needs - Involving all relevant stakeholders in the policy-making process - Support for civil society - Establish a national health equity surveillance system - Build capacity for health equity impact assessment - Build comprehensive package of quality early child development programs and services for children, mothers, and other caregivers - Addressing the provision of quality education for children in preprimary school in general health policies announced by the supreme leader - Economic and social policies - Build universal social protection systems - Universal basic health insurance scheme - Establishing SDH research centers in provincial universities - Build and strengthen national capacity for progressive taxation. - New national and global public finance mechanisms should be developed, including special health taxes and global tax options. - Intersectoral environmental interventions - The culture of cooperation and teamwork - Bureaucratic obstacles - Monitoring these intersectoral action |
| Jalil koohpayehzadeh & Seyyed saeed kassaeian, 2018 [49] | Determining and prioritizing indicators of public health coverage in Iran: A qualitative study |  | Qualitative study | - Political and financial commitment - Public utility services - Civil society organizations - Technical capacity to produce - Use research evidence - Economic growth - Law of compulsory social security of workers - Compulsory health insurance - Implement the rules of the World Health Organization - Expand of country's health care network - The family doctor plan - The transformation plan Health system - Death rate of pregnant women - Vaccination coverage of children under 5 years' old - Life expectancy at birth - Infant mortality rate - Death rate of children under 5 years of age - Death rate of children under 1 years of age - High blood sugar levels in adults - Access to healthy water - Death rates due to cancer - Cesarean delivery ratio - Percentage of health costs from gross domestic product - Out of pocket - The number of manpower in the health sector to the population - Satisfaction of services recipient |
| Letafat, M, et al, 2018 [50] | Universal Health Coverage (UHC) in Iran | Letter to the Editor |  | - Family physician - Referral system - Increased access to first-level service packages - Basic package of services covered by insurance companies - Multiplicity of the insurance funds - Limitation of financial resources - Cost management - Using evidence-based interventions - Policies to create harmony between the insurance companies and the MOHME. - Inadequate budgets - Clear borders between public and private systems - Drastic changes in epidemiology of diseases and demographic characteristics - Lack of accountability to demands of society due to the limitations of manpower - Negligence of social variables - Political commitment from the government part - Change of attitude to the health sector - Making furthermore efforts for reaching an efficient health system |
| Doshmangir, L. et al, 2020 [51] | Determinants of catastrophic health expenditures in Iran: a systematic review and meta‑analysis | Article | A systematic review and meta‑analysis | - Social health insurance - Tax based financing - Mix of prepayment mechanisms - Economic development - Prepayment mechanisms - Protect the poor and disadvantaged - Design a benefits package - Level of cost sharing by the patients. - Catastrophic health expenditures - Out-of-pocket (OOP) |
| Doshmangir, L.et al, 2019 [52] | So Near, So Far: Four Decades of Health Policy Reforms in Iran, Achievements and Challenges | Article | Review | - Advances in public health and medical sciences - Establishment and expansion of health facilities within the hard-to-reach areas aiming to enhance equity in access to needed healthcare services - Domestic production of most medicines and medical equipment - Expansion of health insurance coverage. - Health financing - Protecting the public against high expenditure of medical care - Establishment of referral system and rationalization of service utilization - Provision of high-quality healthcare services to all in need - Conflict of interest in health policy making - Structural and functional reforms - Efforts and enhance intersectoral collaboration to address social determinants of health - Improve actions for prevention and control of non-communicable diseases - Sustainable health financing - Reduce the provincial inequality - Strengthening prepaid-based mechanisms - Equitable distribution of financial resources - Low quality of insurance performance - Strategic purchasing mechanisms - Enhance quality of health care - Community needs-based health education - Enhancing the quality and quantity of health workforce education - Emphasizing public health than medical services - Emphasizing outpatient rather than inpatient health services - Putting more priority on the health of deprived sittings - Increasing financial and administrative authority of medical universities to develop educational and research service - Providing more educational facilities for students - Further intervention by the medical university in providing health services - Integration between the primary, secondary and tertiary levels of service provision - Reducing or eliminating the resilience of staff provincial health organizations in collaboration with the health educational system - Moving towards decentralization in health management across the provinces - Integrating financial resources of health provision and health education - Increasing people's access to health services by providing sustainable financial resources - Encouraging financial autonomy of hospitals and reliance on internal resources - Integration of health information circulation in the health system - Rectifying decision-making processes in different levels of the MOHME - Overlaps in healthcare provision - Management autonomy of hospitals aiming for faster decision-making processes and delivering health services - Increasing competition among health care providers - Enhancing access to health care services - Improving the performance of PHC network - Providing infrastructures for implementing FP plan - Reducing financial burden and OOP expenditure - Strengthening the stewardship function of the MOHME to get more control over financial resources - International unfair sanctions against Iran - Access to essential medicines - Price of medicines - Conflict of interest - Structural reforms - Respond to demographic changes - Redefinition of health insurance system - Ensure sustainable revenue raising - Contribution of the insured - Multisectoral collaboration - Adjust the ever-growing healthcare expenditure - Referral system - Knowledge of the structures of government - Society participation |
| Doshmangir, L.et al, 2019 [53] | Policy analysis of the Iranian Health Transformation Plan in primary healthcare |  |  | - Conflicts of interest - The changes in diseases pattern - Expand the service package of the PHC - Inflexibility of existing initiatives to respond to the changing needs - Policy dynamism - Hasty policy implementation by politicians - He political will - The financial commitments - Improved fiscal capacity of the country - Referral system - Changing health needs and expectations - Changing demographics - Disease burden - Issues of urbanization - Development of high-quality and comprehensive PHC - Engaging independent organizations in result- based monitoring and evaluation system |
| Maleki, M R, et al. 2010 [54] | Sustainable universal health insurance coverage barriers in Iran: 2007 | Article | Qualitative study | - Uncertainty of insured statistics - Non-transparent financial flow - The fragmentation insurance system - Compulsory Insurance - Basic benefit package based on scientific principles - Payment system - Demand control - Managerial efficiency - Systematic perspective - Designing a long-term plan - Scarcity Financial resources - Managerial capacity - Insurance fund management - Overlap in statistics - Government role - Injustice in the payment of obligations - Subsidies targeted - Insurance premiums - Non-scientific policy issues regarding insurance premiums - Out of pocket - Coverage of services - Disruption in the organization of funds - Pay management costs - Standard treatment guidelines - Government commitment - National information system - Managerial instability - Problems of law |
| Meskarpour Amiri, M, et al, 2019 [55] | Health informal payments and their main determinants: The case of Iran |  | cross-sectional and descriptive-analytical study | - Informal payments - Basic health insurance coverage - Awareness of health insurance entitlements - Education - Perceived behavioral control - The pattern of health service utilization - Reduce the financial relationship between physician and patients |
| Kabir, M. J. et al, 2019 [56] | Developing basic health services packages: Defining a prioritization of effectiveness criteria |  | Mixed-method study  (qualitative and  Quantitative study) | - Basic health services packages - Health with priority - Structure and potential capability of service providing system - People demand - Utilization - Financial resources - Social acceptability of service - Obligations and demands of government - Burden of disease - Vulnerable target groups - Community needs |
| Naghdi, S. et al, 2017 [57] | The Barriers to Achieve Financial Protection in Iranian Health System: A Qualitative Study in a Developing Country |  | Qualitative Study | - Inter-sectoral collaboration - Comprehensive benefits package - Cover the poor people - Traditional methods of delivery - Financing and control of health system - Implementation of comprehensive actions within health systems - Out-of-pocket payment   ***Social, political & economic context of country***   - The viewpoints of politicians and governors about financial protection - General political & economic structure of country - Unified & central Stewart for financial protection - NGOs and charities - Health system efficiency - Strengthening control and surveillance in health system   ***UHC dimensions***   - Comprehensive& integrated information system - Disproportionate developing if UHC and its components - Capacity building in a sustainable way - Mobilize resources - Integrate fragmented efforts into one direction to achieve the UHC. - Rate of economic growth - Rate of unemployment - International sanctions against Iran - Domestic mismanagement |
| Nosratnejad, S, et al, 2019 [58] | Universal Health Insurance and the Reasons of not Coverage in Iran: Secondary Analysis of a National Household Survey |  | Survey | - Knowledge of how to insure - Employer's escape from insuring employees or employees - Unemployment of the head of the household - Household income level - Financial ability - Insurance is useless |
| Dehnavieh, R.et at, 2018 [59] | Quality of health services of the Islamic Republic of Iran: Status, barriers and improvement strategies |  | Mixed-method study (qualitative and Review study) | - Commitment of managers to quality - Familiar with the concepts of management and quality - Rapid change of managers - Clear rules for appointing managers - Quality management tools - Evaluators and experts familiar with quality tools - Motivation in personnel - Manpower - There is pessimism about quality tools - Weakness in teamwork - The quality-based incentive and punishment system - High workload in some providers - Distribution of manpower - The health status of providers - Interaction between the deputies of the ministry - Inter-sectoral communications, especially between public and private - Definition of executive structures - Referral system - Provide constructive feedback from evaluation results - Role of insurance in monitoring the quality of services - Teaching the concepts of quality improvement - Non-functional training - Weakness in financing education - Connection between the payment system and the motivation to improve quality - Feelings of low need for change - Hurry to the conclusion - Use of clinical guidelines - A systematic view of error - Knowledge transfer - Quantitative and qualitative indicators and standards - Financial limitations - Access to information - Use patients' electronic health records - Management's poor view of prevention services and the allocation of less facilities to it - System of registration of service statistics and health consequences - Service package - Access to services - The space and equipment of some service providers - Track patients - The distribution of facilities and infrastructure - Supportive rules - Supervision by the ministry and the university |
| Sajadi, H. S. & Majdzadeh, R. 2019 [60] | From Primary Health Care to Universal Health Coverage in the Islamic Republic of Iran: A Journey of Four Decades |  | Review | - Continuity, comprehensiveness and coordinated of care - Required infrastructures for first contact access - Centralized decision-making - The role of private providers was not considered - Adequate financial resources, fair distribution and motivated mechanism for providers' payment - The managed of information system - The providers’ payment mechanism - Adequate facilities and support in disadvantaged areas - Training and capacity-building - Referral system - Health information system - Top-down approach - The active participation of some stakeholders - Coordination among different channels of health service - Respond to changes through a multisectoral approach - Demographic and epidemiologic transitions from the communicable diseases to the ncds. - Redesigning the health service delivery - Integrated people-centered care - The predominance of medicalization and disease-focused interventions made the primary care system weak. - The primary care system - Financial shocks - International sanctions - Political commitment - Sustainable financing - Integrity to provide health care - Unskillful and unmotivated health workforce - Good governance |
| Sajadi, H. S. et al , 2019 [61] | Universal Health Coverage in Iran: where we stand and how we can move forward | Debate |  | - Sustainability of resources - Established and updated service delivery management - Strong governance arrangement - Strong national commitment - Sanctions - Financial protection against health expenses - Increase access to high quality health care and facilities - Improve the performance of the health system - Improve beyond the health sector - Reinforce and institutionalize intersectoral cooperation - Involve the society in improving health - Developing information technology and registration systems in the health sector - Train, empower managers and first level health providers and strengthen their skills - Improve the quality of health services in the public sector - Prevent informal payments for health care - Out of pocket payment for inpatient and outpatient services - Control the price or medicine and medical devices - Manage resources and financial discipline - Health information system - Distribution of health workforce - Resource (human, financial, physical) - Regulate market of medical equipment - Adaptability of health care system, especially in primary health care, to the ongoing changes - Dual practice of physician and other health workforce - Health care utilization - The activity of private sector in the health market - Cost effectiveness health benefit package - The method of providers payment |
| Soltani, S. et al, 2019 [62] | Financial Barriers to Access to Health Services for Adult People with Disability in Iran: The Challenges for Universal Health Coverage |  | Qualitative Study | ***Health insurance***   - Insurance coverage for healthcare services - Coverage for rehabilitation supplies and equipment, i.e. Wheelchairs, walkers, insoles, braces, prosthetic limbs - Copayment for physical therapy, laboratory tests and some medicines   ***Afordability***   - Income of people - Financial support - Low levels of pensions for PWD - Mismatch between the allocation of subsides and the severity of disability and socioeconomic status - The condition of health costs reimbursement   ***Transportation costs***   - High cost of transport to healthcare facilities for PWD |
| Abdi, Z. et al, 2019 [63] | Universal health coverage in Iran: What kind of  knowledge is needed to achieve the goals? | Debate |  | - Health insurance system - Integration of NCD prevention strategy into primary health-care - Identification of cost-effective population-level interventions for NCD and road injury prevention - Epidemiological assessment of NCDs in Iran by geographic areas - Distribution of health resources and services - Investigating the current and the future common health problems in Iran’s elderly - The human resources for UHC research - Unsustainable financing - Inefficiency of the health system - Weakness of governance - Resistance economy in the health sector and operationalizing ite - Prioritizing health services - Family physician program - Dual practice - Referral system |
| Behzadifar M, et al. 2020 [64] | The ‘Health Transformation Plan’ in Iran: A policy to achieve universal health coverage in slums and informal settlement areas | Article | Review | - High‐quality healthcare service packages - Reducing health costs - Financial support - Increasing insurance coverage - Efficient and effective systems and infrastructures - Increasing people's health education - Outsourcing services - Services offered by non‐governmental organisms |
| Doshmangir L, et al. 2021 [65] | Iran health insurance system in transition: equity concerns and steps to achieve universal health coverage | Article | Review | - Health insurance reforms - Health equity by expanding population coverage - Benefits package - Enhancing financial protection - Lack of suitable mechanisms to collect contributions - The compulsory health insurance coverage law is not implemented in full - Gap between private and public medical tariffs - High out-of-pocket health expenditure - Controlling the total health care expenditures - Fragmentation in the pooling of health insurance funds |
| Bastani P, et al. 2020 [66] | Universal health coverage under the Joint Comprehensive Plan of Action’s sanctions:  strategic purchasing approach in the Iranian health system | Article | Qualitative study | - The sanctions - Strategic purchasing - Economic evaluation - Payment system - Priority population |
