# Supplementary material for: The influential factors for achieving universal health coverage in Iran: a multimethod study
Source: BMC Health Serv Res. 2021 Jul 22;21:724. doi: 10.1186/s12913-021-06673-0 (PMC8299681; doi:10.1186/s12913-021-06673-0)
Supplement: Supplementary file 3 — Additional file 3. Appendix 3- Identification affecting factors to achieving UHC trough systematic review in Iran. [file 12913_2021_6673_MOESM3_ESM.docx]

| **Appendix 3.** Identification affecting factors to achieving UHC trough systematic review in Iran | |
| --- | --- |
| - Financial risk protection - The time bounded national action plan - Inter-sectoral communications, especially between public and private - Leadership - Change management - Financial constraints - Health infrastructure - Human resources - Coordination resistance of some organizations - Effective Services Coverage - Training of human resources - Education and Training package for health - Empowering managers and employees of the country's health sector - Empowering people - Creating appropriate atmosphere to criticize findings - Developing a single website for information dissemination and access to required data - Using national media for culture-building - Creating an incentive mechanism - Supporting revision projects and national health indicators development. - Reviewing other countries experiences - Subsidies for prepayments - Tax-based financing - Sustainable financing - Budget as percent of GDP - Out of pocket - Payment exemption - Contribution based on payment capacity - Equity in Access - Political commitment - Financial commitment - Resource production - Capacity building - Management - Prepayment mechanisms - Costs Control - Health insurance system - Focus on poor, disadvantaged and marginalized groups - Family Physician Program - Collect and accumulate - International assistance - Income - Payment systems - The health care technology - Health information system - Quality of health care services - Strengthen the central government's Ministry of Health - Supporting the inhabitancy of physicians in deprived areas - Prioritizing health services - Poverty - Contribution of the insured - The risk of multi aggregation - The economic situation (high or low economic growth) - Government funds to the health sector Research - The high unemployment rate in the country - Conflict of interest - Stability Management - Evidence-based management - Resource management - Distribution of resources - Sustainability of resources - Mobilize resources - Cost management - Catastrophic health expenditures - Benefit package - Control demands - Policies and programs belonging to persons - Private sector - Use of clinical guidelines - Assessment of the status - Primary health care - Development of the country's health care network - Purchasing strategic - Health service tariffs - Weak Parliament - Fragmented health system - Treatment - International sanctions - Referral system - Regular transparency of revenues, expenditures and activities - Provide standards of health care services based on health service leveling - Prevention and control of non-communicable diseases - Prevention center in the current organizational structure | - Perceived behavioral control - Demographic and epidemiologic transitions - Structural barriers in establishing financial communication - Organization's inability to achieve its missions - Systematic evaluation framework - Making appropriate policies on outsourcing - The necessity for grading health service centers and giving the insured sufficient notice of this grading - Integration of insurance organizations - Performance of Supreme Council of Insurance - Absence of obligation for physicians to contract with insurance organizations - Improper use patterns in the health sector - The pattern of health service utilization - Creating an organizational culture - Information and interaction of insurance deductions for heal - Blood transfusion systems - Integrated people-centered care - Provide pre-hospital emergencies to villagers, nomads and less populated cities - Electronic Health Case - Low accountability - Evidence-based policy making - Scientific, transparent, and cost-effective priority-setting - Competitive space between the providers. - Administrative and employment regulation - Accreditation of health system - Reviewing job classification schemes according to the needs of the health system. - Informal payments - Health system efficiency - Developing high-level policy documents based on SDH approach in provincial level called “provincial health plan” - Establishing a community-based center called “neighborhood health center” - Civil society organizations: NGOs and charities - Addressing gender specific health needs - Involving all relevant stakeholders in the policy-making process - Bureaucratic obstacles - Implement the rules of the World Health Organization - Vaccination coverage of children under 5 years old - Life expectancy at birth - Death rate in different group - High blood sugar levels in adults - Negligence of social variables - Structural and functional reforms - Emphasizing outpatient rather than inpatient health services - Moving towards decentralization in health management across the provinces - Management autonomy of hospitals aiming for faster decision-making processes and delivering health services - Access to essential medicines - Policy dynamism - Hasty policy implementation by politicians - Political will - Disease burden - Issues of urbanization - Systematic perspective - Problems of law - Social acceptability of service - Community needs - Weakness in teamwork - executive structures - Feelings of low need for change - Knowledge transfer - Supervision by the ministry and the university - Track patients - Centralized decision-making - Top-down approach - Regulate market of medical equipment - Dual practice of physician and other health workforce - Resistance economy in the health sector and operationalizing ite - Expanding air and ground emergency services coverage - Natural childbirth expansion program - Establishment of health centers - Unconditional care and treatment of the injured in traffic accidents - The presence of specialists in public hospitals - Good governance - Common understanding between stakeholders - Concentration of hospitals in the large metropolises - Overlap in population coverage - Overlaps in healthcare provision - Increase customer satisfaction and service providers - Managerial appointments in health sector - Comprehensive measurements and monitoring of the progress - Different fee schedules practiced by private and public providers |
