# Supplementary material for: The influential factors for achieving universal health coverage in Iran: a multimethod study
Source: BMC Health Serv Res. 2021 Jul 22;21:724. doi: 10.1186/s12913-021-06673-0 (PMC8299681; doi:10.1186/s12913-021-06673-0)
Supplement: Supplementary file 4 — Additional file 4. Appendix 4- Identification affecting factors to achieving UHC trough FGD and semi-structured interviews in Iran. [file 12913_2021_6673_MOESM4_ESM.docx]

| **Appendix 4.** Identification affecting factors to achieving UHC trough FGD and semi-structured interviews in a LMIC | |
| --- | --- |
| - Commitment to access - Equity in access - Financial protection against catastrophic costs - The political and financial commitment - Civil service public - Civil society organizations - Technical capacity to produce - Use of research evidence - of health - Reduce the amount paid by patients in public hospitals - Plan to support the retention of physicians in underserved areas - Financial protection of specific diseases, incurable - Priority services - Distribution of resources - Poverty - Household financial contributions - Income - The risk of multi aggregation - The high administrative costs - Multiplicity of funds - ​​the economic situation (high or low economic growth) - Stability management - The national commitment and political support - Culture and education - Appropriations stable budget from government - Development of clinical guidelines - Integration fund - Family physician program - The level of service in the family physician program - The long-term plan - Assessment of the status quo - Strengthening primary health care - The definition of a service pack - Have political commitment and not having politically look - Financial stability - Purchasing strategic - Legal commitment - Commitment to do - Tariff weak - Lack of accurate information system - Removing political gaps - Lack of insurance - Weak oversight parliament - Functionally independent states - Treatment-centered health system of - Medical education - Setting up referral system (health centers) - Lack of attention to various aspects of economic, cultural and social programs and health interventions - Lack of attention to the structures and substrates in the formulation and implementation of policies | - Fiscal policy space - Capacity - The long-term political commitment - Management - Out of pocket payment - Pre-payment - Costs control - Buy strategically - Financing based on prepayment - Insurance - Mandatory participation inventory salary - Focus on poor and marginalized groups - Development of the family health program - Accumulation and payment - Taxes - Participation mandatory health insurance - Income tax - International assistance - Investment in the development of payment systems - Integration of labor resources in health sector - The network communication facilities and health sector - The health care technology - The information systems in health sector - Quality - Strengthening electronic data recording - Strengthen the central government's ministry - Deductible - Government funds to the health sector - Research - Low per capita income - The high unemployment rate in the country - Inefficient and non-transparent tax system - The low general level of wages of people who pay insurance premiums - The economic crisis - Weaknesses in inter sectoral collaboration - Failure to balance the interests of stakeholders in the health removal and installation of high management levels. - Policies and programs belonging to persons - Collect and accumulate - Due to the position of the private sector in the provision of services - Education and training of human resources in accordance with the needs of the community - Shortage of supply and skyrocketing medical staff - Lack of policy - The lack of evidence-based management - Technology assessment of health - Community-based services - Consistent attention to the treatment and prevention - Lack of public participation system |
