# Supplementary material for: The influential factors for achieving universal health coverage in Iran: a multimethod study
Source: BMC Health Serv Res. 2021 Jul 22;21:724. doi: 10.1186/s12913-021-06673-0 (PMC8299681; doi:10.1186/s12913-021-06673-0)
Supplement: Supplementary file 5 — Additional file 5. Appendix 5- List of excluded studies. [file 12913_2021_6673_MOESM5_ESM.docx]

| **Appendix 5. list of excluded studies** |
| --- |
| 1. Doshmangir L, Sajadi HS, Ghiasipour M, Aboutorabi A, Gordeev VS. Informal payments for inpatient health care in post-health transformation plan period: evidence from Iran. BMC public health. 2020 Dec; 20:1-4.  2. Letafat M, Beyranvand T, Aryankhesal A, Behzadifar M, Behzadifar M. Universal Health Coverage (UHC) in Iran. Iran J Public Health. 2018 Jul;47(7):1061-1062. PMID: 30182014; PMCID: PMC6119564.  3. Rezapour R, Tabrizi JS, Farahbakhsh M, Saadati M, Abdolahi HM. Developing Iranian primary health care quality framework: a national study. BMC Public Health. 2019 Jul 9;19(1):911. doi: 10.1186/s12889-019-7237-8. PMID: 31288783; PMCID: PMC6617563.  4. Bahmanziari N, Takian A. Health system stewardship in Iran: Far from perfect! Med J Islam Repub Iran. 2020 Oct 27; 34:144. doi: 10.34171/mjiri.34.144. PMID: 33437740; PMCID: PMC7787014.  5.Ahmadnezhad E, Murphy A, Alvandi R, Abdi Z. The impact of health reform in Iran on catastrophic health expenditures: Equity and policy implications. Int J Health Plann Manage. 2019 Oct;34(4): e1833-e1845. doi: 10.1002/hpm.2900. Epub 2019 Aug 27. PMID: 31452274. |
