# Supplementary material for: The influential factors for achieving universal health coverage in Iran: a multimethod study
Source: BMC Health Serv Res. 2021 Jul 22;21:724. doi: 10.1186/s12913-021-06673-0 (PMC8299681; doi:10.1186/s12913-021-06673-0)
Supplement: Supplementary file 6 — Additional file 6. Appendix 6- Quality assessment of the study. [file 12913_2021_6673_MOESM6_ESM.doc]

| **Appendix 6.** Quality assessment of the study | |
| --- | --- |
| **Criteria** | **Score (0-3)** |
| Explicit theoretical framework | **3** |
| Statement of aims/objectives in main body of report | **3** |
| Clear description of research setting | **3** |
| Evidence of sample size considered in terms of analysis | **3** |
| Representative sample of target group of a reasonable size | **3** |
| Description of procedure for data collection | **3** |
| Rationale for choice of data collection tool(s) | **2** |
| Detailed recruitment data | **3** |
| Statistical assessment of reliability and validity of measurement tool(s) (Quantitative only) | **-** |
| Fit between stated research question and method of data collection (Quantitative only) | **-** |
| Fit between stated research question and format and content of data collection tool e.g. interview schedule  (Qualitative only) | **3** |
| Fit between research question and method of analysis (Quantitative only) | **-** |
| Good justification for analytic method selected | **3** |
| Assessment of reliability of analytic process (Qualitative only) | **2** |
| Evidence of user involvement in design | **3** |
| Strengths and limitations critically discussed | **2** |
